# Prediction of Maternal Cytomegalovirus Serostatus in Early Pregnancy: A Retrospective Analysis in Western Europe

**DOI:** 10.1371/journal.pone.0145470

**Published:** 2015-12-22

**Authors:** Lorenz Kuessel, Heinrich Husslein, Julian Marschalek, Julia Brunner, Robin Ristl, Theresia Popow-Kraupp, Herbert Kiss

**Affiliations:** 1 Department of Obstetrics and Gynecology, Medical University of Vienna, Vienna, Austria; 2 Section for Medical Statistics, Centre for Medical Statistics, Informatics, and Intelligent Systems, Medical University of Vienna, Vienna, Austria; 3 Department of Virology, Medical University of Vienna, Vienna, Austria; University of Regensburg, GERMANY

## Abstract

**Background:**

Cytomegalovirus (CMV) is the most prevalent congenital viral infection and thus places an enormous disease burden on newborn infants. Seroprevalence of maternal antibodies to CMV due to CMV exposure prior to pregnancy is currently the most important protective factor against congenital CMV disease. The aim of this study was to identify potential predictors, and to develop and evaluate a risk-predicting model for the maternal CMV serostatus in early pregnancy.

**Methods:**

Maternal and paternal background information, as well as maternal CMV serostatus in early pregnancy from 882 pregnant women were analyzed. Women were divided into two groups based on their CMV serostatus, and were compared using univariate analysis. To predict serostatus based on epidemiological baseline characteristics, a multiple logistic regression model was calculated using stepwise model selection. Sensitivity and specificity were analyzed using ROC curves. A nomogram based on the model was developed.

**Results:**

646 women were CMV seropositive (73.2%), and 236 were seronegative (26.8%). The groups differed significantly with respect to maternal age (*p* = 0.006), gravidity (*p*<0.001), parity (*p*<0.001), use of assisted reproduction techniques (*p* = 0.018), maternal and paternal migration background (*p*<0.001), and maternal and paternal education level (*p*<0.001). ROC evaluation of the selected prediction model revealed an area under the curve of 0.83 (95%CI: 0.8–0.86), yielding sensitivity and specificity values of 0.69 and 0.86, respectively.

**Conclusion:**

We identified predictors of maternal CMV serostatus in early pregnancy and developed a risk-predicting model based on baseline epidemiological characteristics. Our findings provide easy accessible information that can influence the counseling of pregnant woman in terms of their CMV-associated risk.

## Introduction

Cytomegalovirus (CMV) is a member of the *betaherpesvirinae* subfamily of herpes viruses. CMV is highly ubiquitous among humans and can cause a wide variety of clinical manifestations; CMV can establish life-long latency or persistence following primary infection.[1] Primary CMV infection in pregnant women can cause mild febrile illness, as well as other nonspecific symptoms; however, CMV infection is clinically asymptomatic in 90% of cases. Non-primary infection is defined as infection with a different strain of CMV or reactivation of latent virus with pre-existing antibodies; non-primary infection generally does not cause maternal symptoms.[2]

Among women of reproductive age, the prevalence of antibodies in the serum (i.e., seropositivity) due to prior CMV exposure ranges from 45% in developed countries to 100% in developing countries and is associated with several epidemiological factors, including age, gravidity, parity, place of birth, and socioeconomic status.[3–6] Because seroprevalence rates can reflect the size of the virus reservoir, maternal serostatus can have an impact on the incidence of congenital CMV infection.[7] CMV is the most prevalent congenital viral infection, affecting 0.64% of newborn infants.[8, 9] However, this prevalence varies widely among study populations; For example, in Europe, a highly industrialized region with relatively low overall maternal CMV seroprevalence, regional CMV seroprevalence ranges from as low as 0.1% to as high as 2%.[8] In developing countries with high rates of maternal CMV seropositivity, even higher rates (1–5.4%) of congenital CMV prevalence have been reported.[10, 11]

CMV can be transmitted vertically through intrauterine infection, peripartal transmission, cervicovaginal secretions during vaginal delivery, or breastfeeding. Because cytotrophoblasts in the placenta are permissive to CMV replication, the most common route of vertical transmission is infection of the placenta and subsequent transmission to the fetus, where the virus can infect multiple tissues.[12] Clinical congenital disease can include features such as small for gestational age, microcephaly, ventriculomegaly, chorioretinitis, hepatitis, splenomegaly, thrombocytopenia, and petechiae; newborns with this disease have a mortality rate of approximately 5%.[13] Moreover, approximately 50% of survivors develop severe long-term neurological deficits, including progressive hearing loss and/or cognitive impairment.[14]

Most cases of symptomatic congenital disease are caused by primary maternal CMV infection.[15] The rate of fetal infection ranges from 33% to 75%, and the prevalence of disease can reach 50% among primary infections that occur within the first half of pregnancy; In contrast, non-primary infections are transmitted intrauterine in only approximately 1% of cases, and more than 90% of infected infants are healthy.[16, 17] However, a growing body of evidence suggests that non-primary infections may also constitute a significant cause of severe congenital CMV disease and can contribute significantly to the global disease burden associated with congenital CMV exposure.[7, 18–20]

Nevertheless, the presence of maternal antibodies to CMV due to CMV exposure prior to pregnancy is the most important protective factor against congenital CMV disease.[4] Worldwide, seropositivity rates are lowest in Western Europe and the United States of America.[21] This low rate is associated with a higher risk of primary CMV infection during pregnancy.

Despite the extraordinary disease burden that primary CMV infection during pregnancy places on the newborn infant, routine serological screening of pregnant women for CMV is not currently recommended.[22] Nevertheless, several options are available to prevent fetal CMV infection during pregnancy. Confirming CMV seronegativity and educating women during pregnancy can help modify maternal behavior and can decrease the rate of seroconversion in pregnant women who are at risk of infection.[23–25] Hyperimmunoglobulin therapy in pregnant women with primary CMV infection is an interesting—albeit experimental—approach that may reduce the rate of congenital infections in selected cases, even though a recent randomized, placebo-controlled trial failed to confirm the initially promising results.[17, 26, 27]

Identifying women who are at risk for seronegativity during pregnancy and understanding the resulting risk of subsequent primary CMV infection during pregnancy are crucial steps towards preventing congenital CMV infection. The aim of this study was to identify potential predictors, and to develop and evaluate a risk-predicting model for the maternal CMV serostatus in early pregnancy.

## Materials and Methods

### Participants

From December 2009 through April 2013, pregnant women who were receiving routine prenatal care at the Department of Obstetrics and Fetomaternal Medicine at the Medical University of Vienna were invited to participate in Biotest study 963, a randomized, open, controlled, prospective, multicenter and multinational study (study title: “Prevention of Congenital Cytomegalovirus Infection in Infants of Mothers with Primary Cytomegalovirus Infection during Pregnancy”). The inclusion criteria were gestational age ≤13 weeks + six days and maternal age 18–45 years. At the initial screening visit of the study, CMV-specific antibodies were measured, and the results were documented in the patient’s medical file. All participating subjects provided written informed consent.

For the present study, we performed a retrospective chart analysis of all patients who were eligible for the Biotest study at our study site. Data regarding maternal CMV serostatus, demographics, maternal and paternal migration background, and educational level were extracted from the medical files. Since November 2010, this parental background information has been collected routinely during medical interviews conducted at the first prenatal visit at our department. Women who were seropositive for CMV IgM antibodies and women for whom more than one variable was missing were excluded from the analysis. This study was approved by the institutional review board of the Medical University of Vienna (Reference number 1704_2013).

### Definition of terms

**Fig 1:**
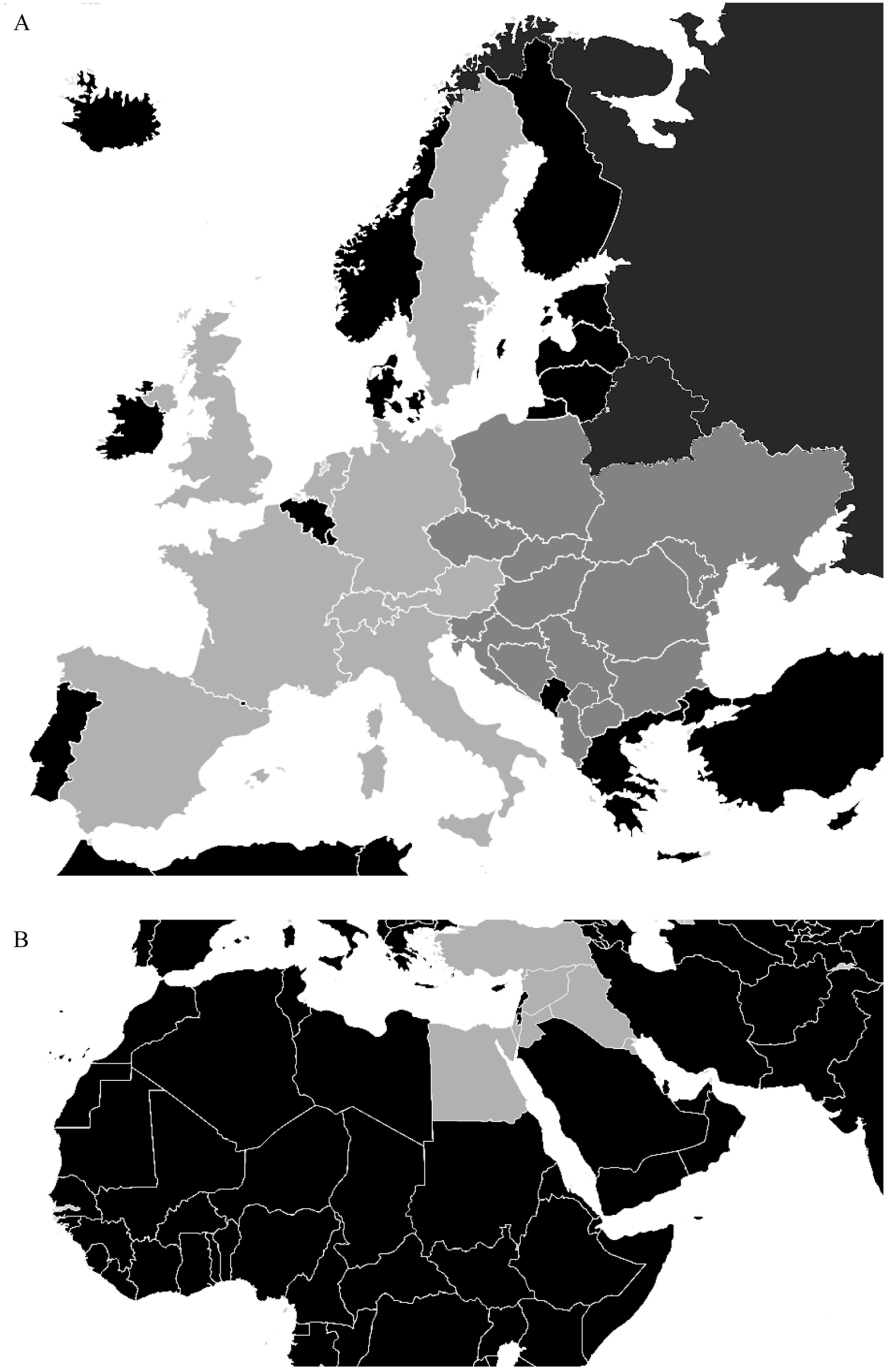
Regions of origin by place of birth of included patients. (A) Light gray: Western Europe; Dark gray: Eastern Europe. (B) Light gray: Middle East.

Seropositivity was defined as the presence of CMV-specific IgG antibodies in the maternal serum; the presence of this antibody serves as a marker for whether the woman has ever been infected with CMV. Seronegativity was defined as the absence of CMV-specific IgG antibodies. Seroprevalence was defined as the prevalence of CMV seropositivity within a defined population. Gravidity refers to the total number of previous pregnancies; Parity refers to the number of viable previous pregnancies. Maternal and paternal educational status (ES) was classified as the completion of no education, primary education, or lower secondary education (ES 1); upper secondary education (ES 2); post-secondary non-tertiary education (ES 3); or tertiary education (ES 4). Migration background (MB) was used to define an individual who was born outside of Western Europe and is currently living in (i.e., emigrated to) Western Europe. The various migrational regions are illustrated in Fig 1.

Because a generally accepted definition of “Western Europe” is not available, we divided Europe into Western Europe and Eastern Europe based on the former Iron Curtain, which was a political and physical boundary that divided Europe into two separate regions until 1991, including emigration restrictions.

### Statistics

All analyses were performed using the statistical software package R, version 3.1.

Maternal age is reported as mean (+/- standard deviation); all other variables were categorical and are reported as absolute or relative frequencies. For comparisons of distributions between groups, the Student’s *t*-test was used for maternal age; the chi-square test was used for all other variables. P-values ≤ 0.05 were considered statistically significant. To describe the correlation between parameters, Spearman’s rank correlation coefficient was calculated. To predict serostatus, a multiple logistic regression model was generated by stepwise forward-backward model selection starting from a null-model and using the Akaike information criterion as the selection criterion.[28] The scope of possible predictor variables included maternal age, gravidity, parity, use of assisted reproduction techniques (ART), ES, and MB, as well as paternal ES and MB. In addition, all pairwise interactions were permitted to be selected in the model. The model’s sensitivity and specificity for various cut-off values were calculated using receiver operating characteristic (ROC) curves. To assess the predictive quality of the selected model in a new data set, five-fold cross-validation was performed. The data set was divided randomly into five subsets of approximately equal sample size. For cross-validation, the model coefficients were estimated from the five possible estimation data sets, each of which contained four out of the five partial data subsets. The resulting models were then used to predict serostatus in the respective prediction data set that was not used for the estimation. For each prediction data set, an ROC curve was calculated, and the area under each ROC curve (AUC) was compared to the AUC of the original model.

This model allows us to predict maternal CMV serostatus using the set of selected variables. A score is calculated as the sum of the regression coefficients that match a woman’s observed variable values, age times the regression coefficient for age and the model intercept. This score is transformed to a predicted probability using the inverse logit link function. Thus, the following equation was used to calculate the probability of seropositivity (*Prob*):

$$Prob\;=\;\frac{e^{Score}}{1+e^{Score}}$$

To facilitate calculation of a predicted probability for seropositivity using the logistic regression model, a nomogram was developed. In the nomogram, probability of CMV seropositivity can be determined by reading points for each variable from the matching lower scale, summing the points, and identifying the prediction of seropositivity associated with the total points line.

Note that the nomogram sum score is 1.11 larger than the score directly derived from the model coefficients as the intercept is accounted for implicetely in the nomogram.

## Results

### Descriptive statistics

From a total of 998 women who were screened for the Biotest study since November 2010, the complete data set (or only one variable missing) was available for 882 women; these 882 patients were included in our final analysis. The characteristics of these patients are summarized in Table 1. The participants were then divided into two groups based on their serostatus; 646 women were seropositive (73.2%), and 236 were seronegative (26.8%). Based on bivariate analyses, these two groups differed significantly with respect to maternal age, gravidity, parity, the use of assisted reproduction techniques, maternal and paternal migration background, and maternal and paternal education level (Table 1)

**Table 1:** Summary of CMV-seronegative and CMV-seropositive women in early pregnancy.

	N	Total sample	CMV seronegative	CMV seropositive	*p*-value	OR (95% CI)
Total	882	882 (100)	236 (26.8)	646 (73.2)		
Maternal Age, years	882	30.63 ± 5.49	31.49 ± 5.51	30.32 ± 5.46	0.006	
Gravidity	881				<0.001	
1		291 (33)	104 (36)	187 (64)		1 (reference)
2		276 (31)	81 (29)	195 (71)		1.3 (0.9–1.9)
3		184 (21)	35 (19)	149 (81)		2.4 (1.5–3.7)
≥4		130 (15)	16 (12)	114 (88)		4.0 (2.2–7.1)
Parity	881				<0.001	
0		398 (45)	144 (36)	254 (64)		1 (reference)
1		301 (34)	70 (23)	231 (77)		1.9 (1.3–2.6)
2		122 (14)	16 (13)	106 (87)		3.8 (2.1–6.6)
≥3		60 (7)	6 (10)	54 (90)		5.1 (2.1–12.2)
Assisted Reproduction	878				0.018	
No		837 (95)	217 (26)	620 (74)		1 (reference)
Yes		41 (5)	18 (44)	23 (56)		0.5 (0.2–0.8)
Origin by place of birth (M)	882				<0.001	
Western Europe		432 (49)	204 (47)	228 (53)		1 (reference)
Eastern Europe		273 (31)	23 (8)	250 (92)		9.4 (6.1–15.5)
Middle East		85 (10)	3 (4)	82 (96)		24.5 (7.6–78.6)
Others		92 (10)	6 (7)	86 (93)		12.8 (5.5–30.0)
Migration Background (M)	882				<0.001	
No		433 (49)	204 (47)	229 (53)		1 (reference)
Yes		449 (51)	32 (7)	417 (93)		11.6 (7.7–17.4)
Migration Background (P)	864				<0.001	
No		449 (52)	196 (44)	253 (56)		1 (reference)
Yes		415 (48)	33 (8)	382 (92)		9.0 (6.0–13.4)
Migration Background (combined)	864				<0.001	
M: No P: No		349 (40)	177 (51)	172 (49)		1 (reference)
M: No P: Yes		74 (9)	21 (28)	53 (72)		2.6 (1.5–4.5)
M: Yes P: No		100 (12)	19 (19)	81 (81)		4.4 (2.6–7.5)
M: Yes P: Yes		341 (39)	12 (4)	329 (9)		28.2 (15.3–52.1)
Education Status (M)	855				<0.001	
ES 1		142 (17)	15 (11)	127 (89)		1 (reference)
ES 2		360 (42)	77 (21)	283 (79)		0.4 (0.2–0.8)
ES 3		92 (11)	31 (34)	61 (66)		0.2 (0.1–0.5)
ES 4		261 (31)	104 (40)	157 (60)		0.2 (0.1–0.3)
Education Status (P)	827				<0.001	
ES 1		128 (15)	17 (13)	111 (87)		1 (reference)
ES 2		387 (47)	85 (22)	302 (78)		0.5 (0.3–1.0)
ES 3		37 (4)	16 (43)	21 (57)		0.2 (0.1–0.5)
ES 4		275 (33)	107 (39)	168 (61)		0.2 (0.1–0.4)

Maternal age is presented as mean (SD), the other variables as numbers (%).

M indicates maternal; P, paternal; OR, odds ratio versus the reference group; CI, 95% confidence interval.

In general, the rate of seropositivity increased with increasing gravidity. The correlation between gravidity and parity was high (r = 0.80). Women who were born in Western Europe had significantly lower seroprevalence than women who were born outside of Western Europe (p<0.001). Because no difference was observed with respect to seroprevalence between different regions outside of Western Europe, we collapsed the information regarding origin into a binary variable with values MB (for participants who migrated to Western Europe) or no MB (for participants who were born in Western Europe).

Similarly to maternal MB, paternal MB was also associated with seroprevalence (p<0.001). Seroprevalence was highest (96%) among women with both maternal and paternal MB. The various countries of origin and the number of included patients from each country are summarized in S1 Fig.

Low maternal and/or paternal ES was associated with higher seroprevalence (*p*<0.001); moreover, maternal ES and paternal ES were significantly correlated (r = 0.63). The use of ART was inversely correlated with seropositivity. We also found an inverse correlation between maternal age and seropositivity. This latter finding appears to be contradictory to our finding that increasing gravidity is associated with higher seroprevalence; we therefore compared women with MB (n = 449; 50.9%) with women without MB (n = 433; 49.1%). The results of these two subgroups are summarized in Table 2.

**Table 2:** Comparison between women without and women with migration background (MB).

	N	MB No (433)	MB Yes (449)	*p*-value
CMV Serostatus	882			<0.001
Seronegative		204 (47)	32 (7)	
Seropositive		229 (53)	417 (93)	
Maternal Age, years	882	30.6 ± 5.7	30.7 ± 5.3	0.850
Gravidity	881			<0.001
1		188 (43)	103 (23)	
2		139 (32)	137 (31)	
3		72 (17)	112 (25)	
≥4		34 (8)	96 (21)	
Parity	881			<0.001
0		247 (57)	151 (34)	
1		133 (31)	168 (38)	
2		36 (8)	86 (19)	
≥3		17 (4)	43 (10)	
Assisted Reproduction	878			0.040
No		403 (94)	434 (97)	
Yes		27 (6)	14 (3)	
Migration Background (P)	864			<0.001
No		349 (83)	100 (23)	
Yes		74 (17)	341 (77)	
Education Status (M)	855			<0.001
ES 1		38 (9)	104 (24)	
ES 2		178 (42)	182 (42)	
ES 3		53 (13)	39 (9)	
ES 4		153 (36)	108 (25)	
Education Status (P)	827			<0.001
ES 1		44 (11)	84 (21)	
ES 2		184 (44)	203 (50)	
ES 3		20 (5)	17 (4)	
ES 4		170 (41)	105 (26)	

Maternal age is presented as mean (SD), the other variables as numbers (%).

M indicates maternal; P, paternal.

The women with MB had a seroprevalence rate of 93% compared to 53% in the women without MB. No significant difference was found between the two groups with respect to maternal age (*p* = 0.850, 95%CI for the difference of means: -0.80–0.66). However, both gravidity and parity were significantly higher among the women with MB compared to the women without MB. Moreover, both maternal and paternal ES were significantly lower in the patients with MB (*p*<0.001). Furthermore, the use of ART was less common among the patients with MB (*p* = 0.040)

### Prediction model

To generate a prediction model, a multiple logistic regression analysis was performed in order to identify variables that were predictive of maternal CMV serostatus. Our procedure yielded a logistic regression model for serostatus that includes the following variables: maternal age, parity, maternal ES, maternal MB, paternal MB, and the interaction between maternal MB and paternal MB. To describe the association between MB and serostatus, we set MB as a categorical variable with four stages corresponding to the four possible combinations of maternal and paternal MB. The results of the parameterization of the selected prediction model are summarized in Table 3.

**Table 3:** Selected prediction model for CMV seropositivity.

	Estimate	SE	OR	95% CI	*p*-value^a^
Intercept	1.536	0.597			
Age	-0.042	0.019	0.96	0.92–0.99	0.025
Parity					0.041
0	0		1		
1	0.363	0.207	1.44	0.96–2.16	
2	0.617	0.336	1.85	0.96–3.58	
≥3	1.136	0.510	3.11	1.15–8.47	
Education status (M)					0.039
ES 1	0		1		
ES 2	-0.210	0.342	0.81	0.41–1.58	
ES 3	-0.613	0.408	0.54	0.24–1.21	
ES 4	-0.770	0.361	0.46	0.23–0.94	
Migration Background					<0.001
M: No P: No	0		1		
M: No P: Yes	0.756	0.290	2.13	1.21–3.76	
M: Yes P: No	1.326	0.287	3.77	2.15–6.61	
M: Yes P: Yes	3.101	0.317	22.22	11.94–41.36	

Estimate and SE refer to the model regression coefficients and their standard error, respectively. OR indicates odds ratios when comparing a given class to the reference class of the same variable and holding constant all other variables.

M indicates maternal; P, paternal.

^**a**^Wald test

**Fig 2:**
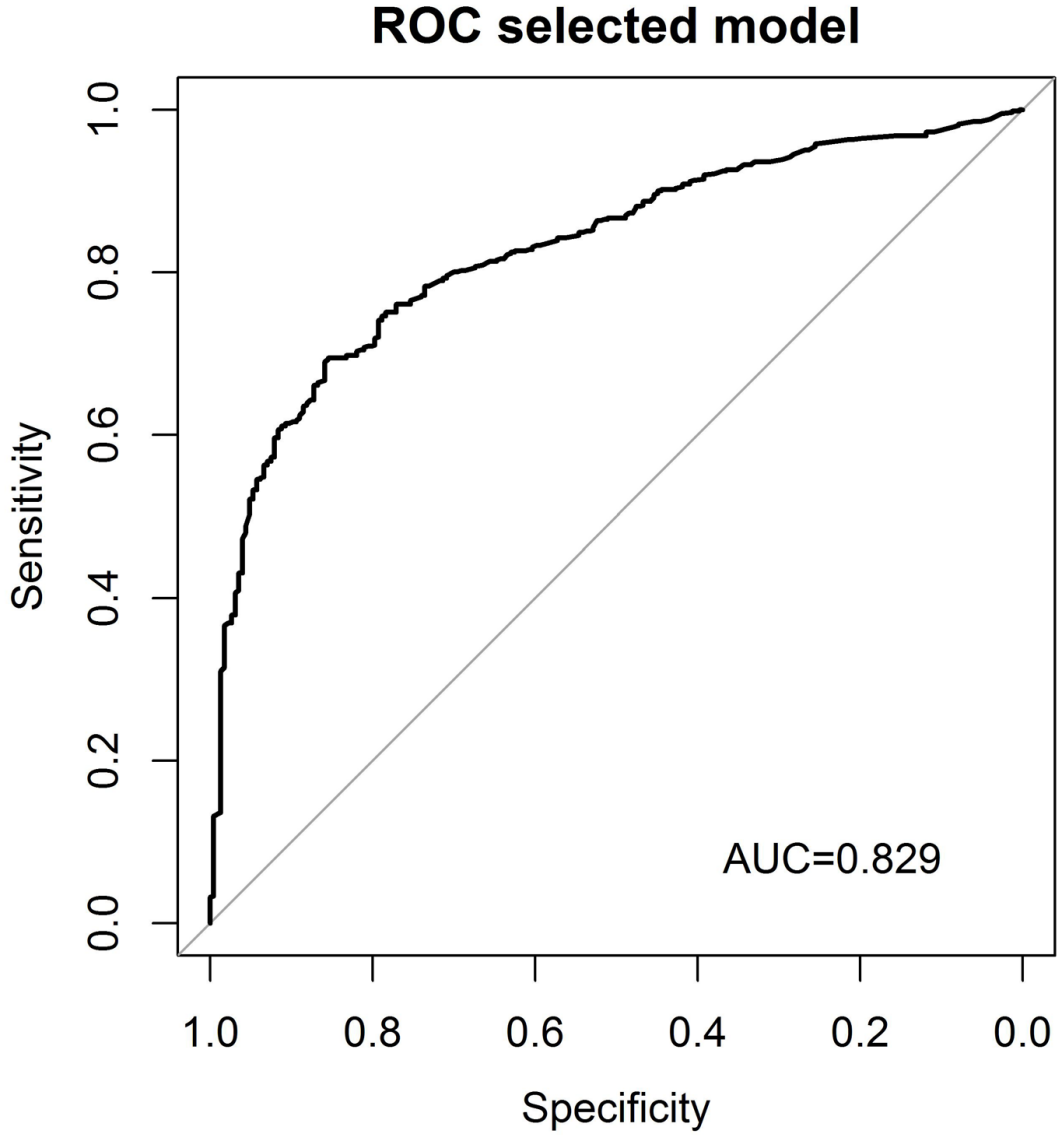
ROC curve for the multiple logistic regression model for CMV serostatus using a set of selected predictors.

The ROC curve for the model based on the complete data set is shown in Fig 2. The highest sum of sensitivity and specificity is obtained when predicting a seropositive status for calculated probabilities above a cut-off of 0.74, with sensitivity and specificity values of 0.69 and 0.86, respectively, for predicting seropositivity. The AUC is 0.83 (95% CI: 0.8–0.86).

In addition, we performed five cross-validation rounds within the data set. The respective AUCs are summarized in Table 4.

**Table 4:** AUCs measured from the five-fold cross-validation analysis.

	AUC	95% CI
Selected model	0.829	0.800–0.857
Cross-validation set 1	0.831	0.768–0.894
Cross-validation set 2	0.774	0.702–0.847
Cross-validation set 3	0.814	0.742–0.885
Cross-validation set 4	0.830	0.771–0.889
Cross-validation set 5	0.850	0.792–0.908

The ROC curves obtained (S2 Fig) and the AUC values suggest that the predictive capacity of the model is stable when used to test a new data set from a similar population.

**Fig 3:**
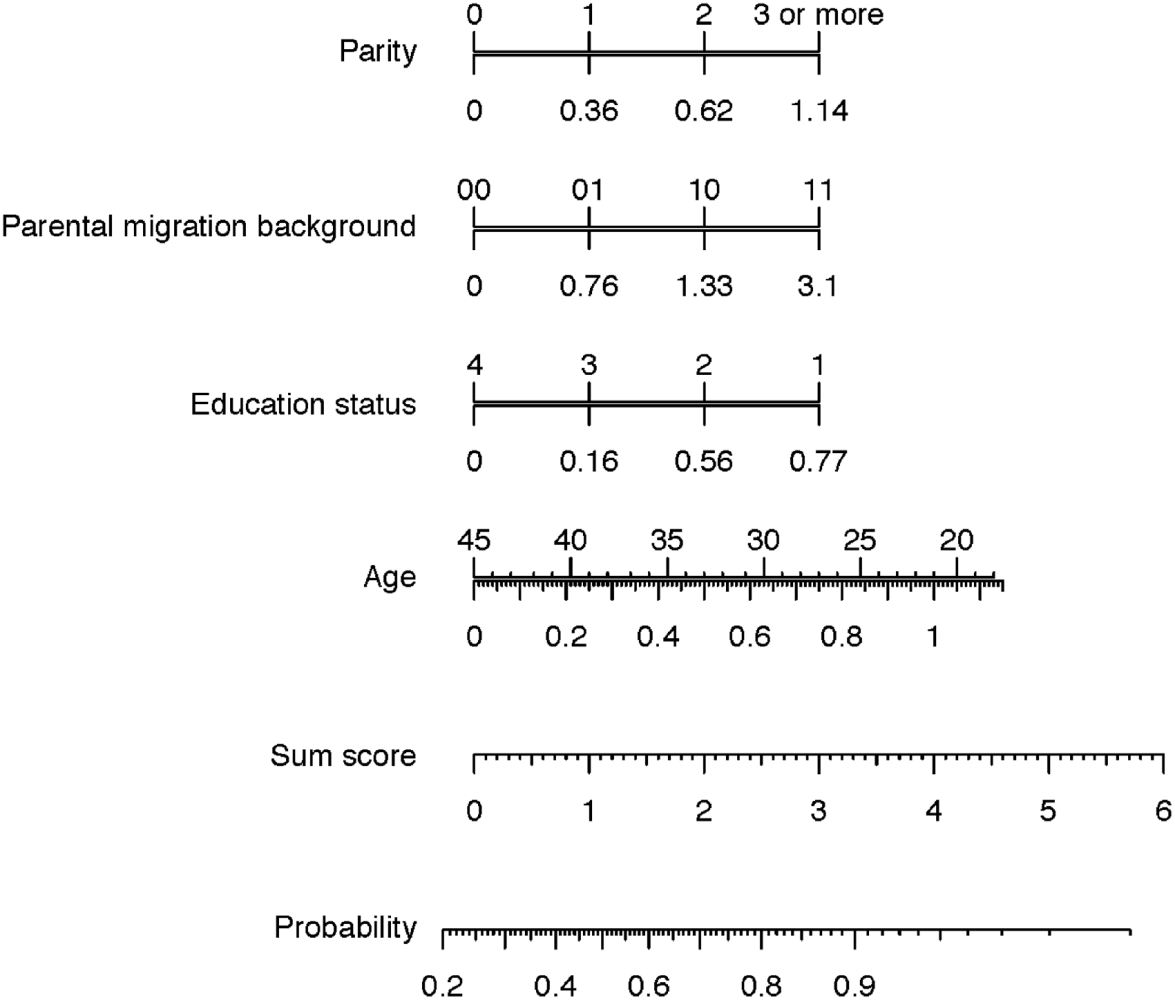
Nomogram to predict maternal CMV seropositivity in early pregnancy. Points for each variable are read from the matching lower scale. The sum of the points plotted on the sum score line corresponds with the prediction of maternal CMV seropositivity, which is assigned by drawing a vertical line to the probability scale. Parental Migration Background (MB) is indicated in four classes: 00, both parents have no MB; 01, only the father has MB; 10, only the mother has MB; 11 both parents have MB. Education status indicates maternal Education Status as defined above.

The resulting nomogram is shown in Fig 3.

## Discussion

Our aim was to identify potential predictors of maternal CMV serostatus in early pregnancy and to create and evaluate a model for predicting CMV serostatus. Knowledge about predictors of maternal CMV serostatus can help to assess a women’s individual CMV-associated risk in pregnancy, influence the counseling of pregnant women, and contribute to efforts to avoid congenital CMV infections. In this study we found that *i*) serostatus is significantly correlated with maternal age, gravidity, parity, and education level; *ii*) maternal MB is associated with higher CMV seroprevalence; and *iii*) paternal MB and ES are correlated with maternal CMV serostatus. Based on these findings, we generated a model for predicting maternal CMV serostatus; in addition, we generated a nomogram that may provide more structured information that can be used to counsel pregnant women regarding the risks associated with CMV seronegativity.

The presence of CMV-specific IgG antibodies in the serum—which indicates a prior or current CMV infection—has been correlated previously with several epidemiological factors, including age, gravidity, parity, and socioeconomic status.[3, 4, 6, 21] Most of these previous findings are consistent with our own findings. However, we found an inverse correlation between maternal age and seropositivity, which is in contrast to other published findings.[4] There are several possible explanations for this discrepancy. Although maternal age was the same in women with MB and women with non-MB, gravidity and parity were higher in the women with MB. CMV can be transmitted via several routes, including adult-to-child, child-to-adult, and adult-to-adult. Most children in developing countries are infected with CMV by the age of three years.[29] Therefore the risk to acquire a CMV infection increases with the number of children living in a family and depends on the child’s care situation.[30, 31] In addition, both maternal and paternal ES were lower in the patients with MB. Lower ES is associated with lower financial income, which results in narrow living space and possibly lower standards of hygiene. This further increases the chance of CMV transmission between mother and children. Lastly, women with MB had more often partners with MB compared to women without MB. Reports of CMV in semen, saliva, and cervical secretions suggest that transmission can occur during sexual activity, and sexual activity can affect CMV seroprevalence among women of childbearing age.[32, 33] Therefore, a woman’s CMV serostatus can be influenced by her partner’s behavior and/or characteristics.

In light of these findings, in our cohort the socioeconomic situation appears to supersede the effect of maternal age on CMV serostatus in early pregnancy. The majority of women born within Western Europe in recent decades have high socioeconomic status, and the average age at their first pregnancy is increasing. We therefore hypothesize that improved socioeconomic conditions in Western Europe have led to a decreased rate of CMV seropositivity, thereby increasing the risk of primary CMV infection during pregnancy.

The Centers for Disease Control and Prevention (CDC) in Atlanta, GA recommend: *i*) against routine serological screening for CMV, *ii*) that women consult their doctor regarding the risk of CMV infection during pregnancy, and *iii*) that pregnant women receive counseling regarding simple hygiene precautions to prevent CMV infection.[22] This approach advocated by the CDC can clearly cause a dilemma for both consulting healthcare professionals and pregnant women—on the one hand, the individual risk of primary CMV infection is difficult to assess in developed countries; on the other hand, the success of preventive measures depends on the woman’s motivation to follow these hygiene recommendations. Given this dilemma, identifying women who are at risk for seronegativity is crucial. Therefore, we attempted to develop a risk-predicting model for maternal CMV serostatus in early pregnancy. Our logistic regression model includes maternal age, parity, ES, maternal and paternal MB, and the interaction between maternal and paternal MB. Because gravidity is correlated with parity, and because paternal ES is correlated with maternal ES, it is not surprising that these variables were not selected in the final model. With sensitivity and specificity values of 69% and 86%, respectively, our method provides a high probability of accurately predicting CMV serostatus in early pregnancy. Although easy accessible to healthcare providers, this information can have a strong influence on the counseling given to pregnant woman in terms of CMV-associated risk.

Our study has several limitations that bear mentioning. First, the women included in the study reflect a group of patients derived from a single tertiary care centre located in a European capital city. Nevertheless, the observed prevalence of CMV seropositivity among the Western European women in our study population is consistent with other published reports.[21, 34] In addition, the usefulness of the presented prediction model appears to be geographically restricted. Although our cross-validation results suggest that the predictive capacity of our prediction model and nomogram is stable, external validation with comparable populations is needed before our model can be extrapolated for use on a broader scale.

A strength of our study is that we analyzed women who were included in the screening phase of a prospective, randomized trial, thus reducing the risk of selection bias that often occurs with retrospective chart reviews. Moreover, the wide range of evaluated predictors including paternal variables is an additional strength of our study.

To conclude, identifying women who are at risk for CMV seronegativity in early pregnancy is important in order to avoid congenital CMV infection. We ascertained predictors of maternal CMV serostatus in early pregnancy and developed a risk-predicting model based on baseline epidemiological characteristics. Our findings provide easily accessible information that can strongly influence the counselling given to pregnant woman in terms of their CMV-associated risk.

## Supporting Information

S1 Fig Regions of origin by place of birth, and the number of included patients. (A) Light blue: Western Europe; Dark blue: Eastern Europe. (B) Light blue: Middle East. (C) Other geographical regions.
(TIF)

S2 Fig ROC curves for the five cross-validation rounds of the presented predictive model. (PDF)
