# Supplementary figures and images for: Prediction of Maternal Cytomegalovirus Serostatus in Early Pregnancy: A Retrospective Analysis in Western Europe
Source: PLoS One. 2015 Dec 22;10(12):e0145470. doi: 10.1371/journal.pone.0145470 (PMC4687935; doi:10.1371/journal.pone.0145470)

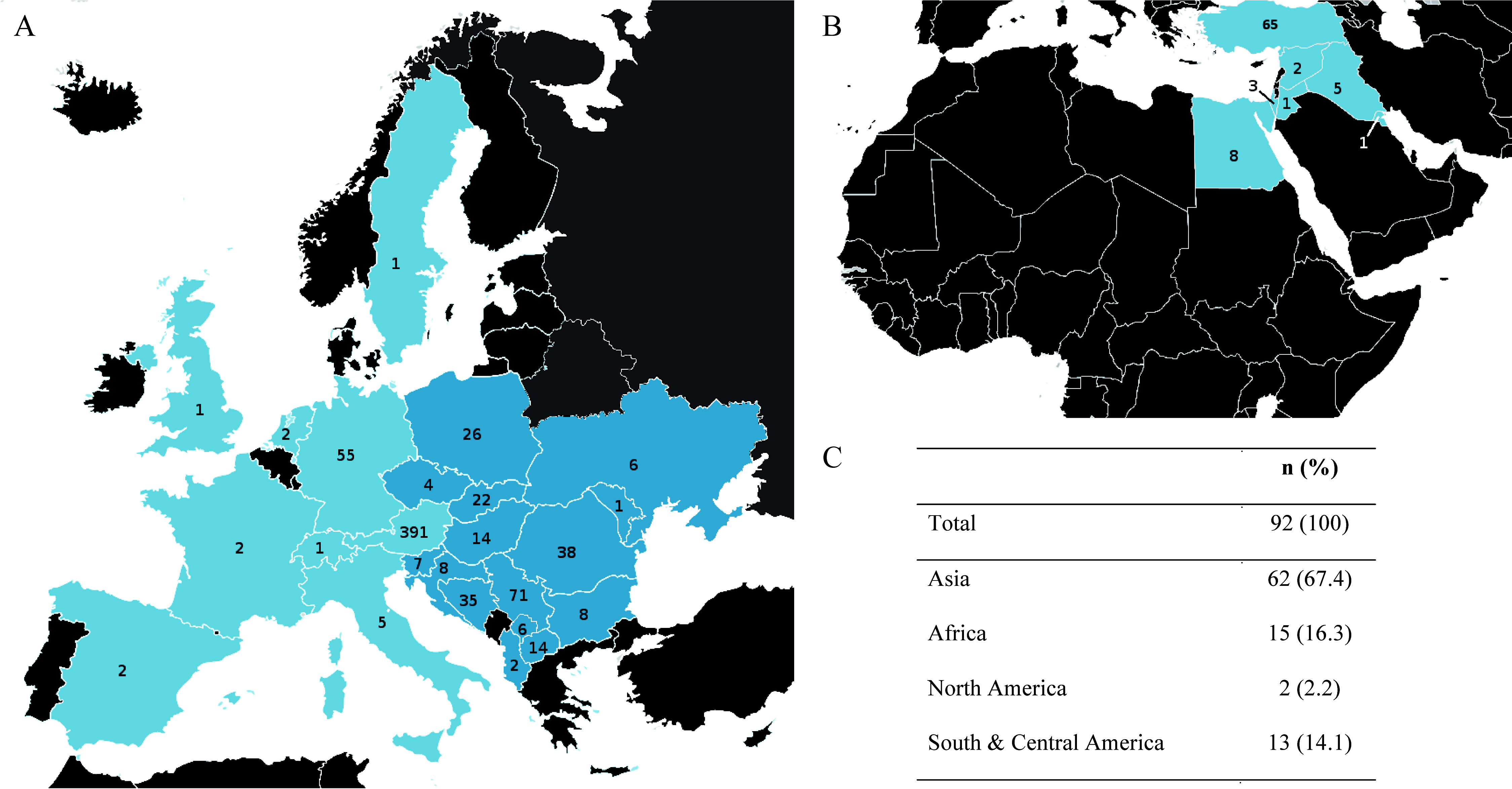

Supplement: S1 Fig — (A) Light blue: Western Europe; Dark blue: Eastern Europe. (B) Light blue: Middle East. (C) Other geographical regions. (TIF) [file pone.0145470.s001.tif]

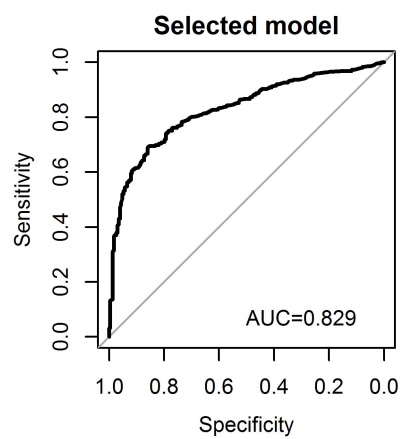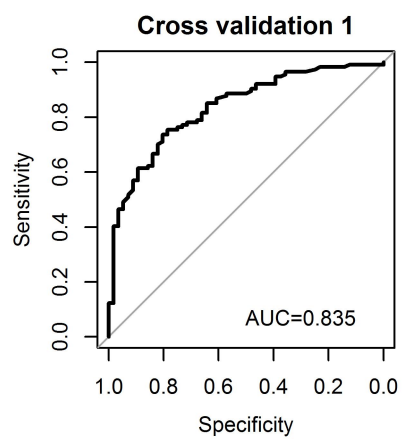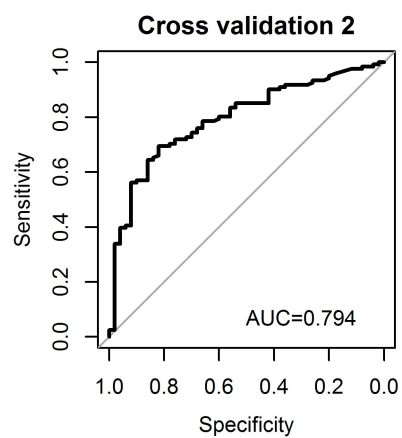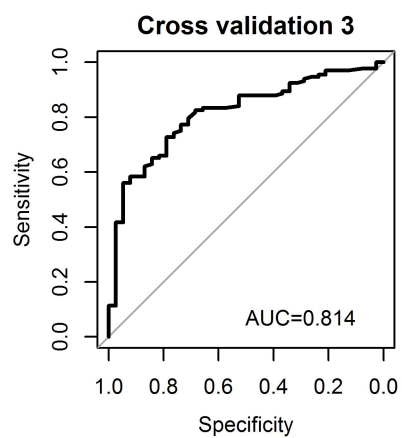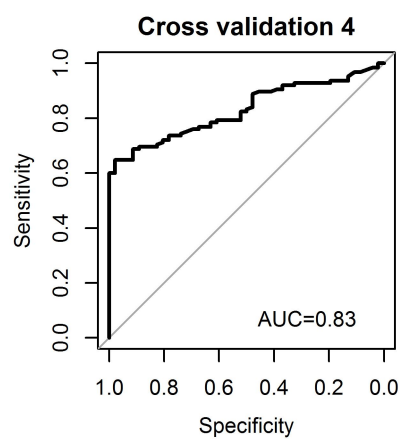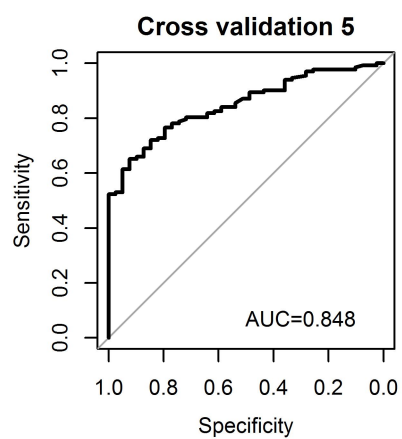

Supplement: S2 Fig — (PDF) [file pone.0145470.s002.pdf]
